# X-ray computed tomography for non-invasive dendrochronology reveals a concealed double panelling on a painting from Rubens’ studio

**DOI:** 10.1371/journal.pone.0255792

**Published:** 2021-08-27

**Authors:** Marta Domínguez-Delmás, Francien G. Bossema, Jan Dorscheid, Sophia Bethany Coban, Moorea Hall-Aquitania, K. Joost Batenburg, Erma Hermens

**Affiliations:** 1 Department of History of Art, University of Amsterdam, Amsterdam, Netherlands; 2 Department of Conservation and Science, Rijksmuseum, Amsterdam, Netherlands; 3 DendroResearch, Wageningen, Netherlands; 4 Computational Imaging Group, Centrum Wiskunde & Informatica, Amsterdam, Netherlands; 5 Leiden Institute of Advanced Computer Science, Universiteit Leiden, Leiden, Netherlands; Chinese Academy of Sciences, CHINA

## Abstract

Dating the wood from historical art objects is a crucial step to ascertain their production time, and support or refute attribution to an artist or a workshop. Dendrochronology is commonly used for this purpose but requires access to the tree-ring pattern in the wood, which can be hindered by preparatory layers, polychromy, wax, or integrated frames. Here we implemented non-invasive dendrochronology based on X-ray computed tomography (CT) to examine a painting on panel attributed to Rubens’ studio and its presumed dating around 1636 CE. The CT images achieved a resolution of 37.3 micron and revealed a double panelling, which was concealed by oak strips covering all four edges. The back (visible) board is made of deciduous oak (*Quercus* subg. *Quercus*), the most common type of wood used in 17th-century Netherlandish workshops, and was dated *terminus post quem* after 1557 CE. However, the front (original) board used for the painting has been identified through examination of the wood anatomy as a tropical wood, probably *Swietenia* sp., a species seldom used in Netherlandish paintings, and remains undated. Its very presence attests the global character of 17th-century trade, and demonstrates the use of exotic species in Flemish studios. The date of the oak board refutes previous results and suggests that this board was trimmed to meet the size of the tropical one, having been glued to it for conservation purposes or with deceiving intentions to pretend that the painting was made on an oak panel. These revelations have opened new lines of art historical inquiry and highlight the potential of X-ray CT as a powerful tool for non-invasive study of historical art objects to retrieve their full history.

## Introduction

A wide array of sciences and techniques are presently available to investigate the production and state of conservation of paintings on panel (e.g. [1]). Establishing the time and place of their production supports their attribution to workshops and artists, whereas the scientific assessment of their condition informs decisions on conservation and restoration treatments. Dendrochronology is the science commonly used to date the wood of panel paintings and establish its provenance. The first tree-ring studies on panel paintings took place in the 1960s [2]. Since then, a wealth of information has been gained about the scale of the north-European timber trade, the use of different wood species for painting supports, the evolution of wood-working and conservation techniques since the late Middle Ages, seasoning time, and the economy of wood resources in European workshops (e.g. [3–9]). Case studies often provide exciting finds, such as timber supply areas that had not been reported before, suggesting a disruption of traditional trade routes in specific years, cheaper transport costs, or even tailor-made trade alliances [10].

Dendrochronological research on panel paintings is done on the transverse ends (end grain) of the boards making up the panel. It usually requires cleaning a thin linear area along the surface with a scalpel blade, micro-abrasive blasting or other methods to remove leftovers of preparatory layers, varnish, or dirt that hamper the visualization of the tree rings [11, 12]. Such invasive procedures are undesirable, as they leave irreversible traces on the objects examined. Therefore, cleaning methods must be carefully considered for each object and set against the possible knowledge gained through the research. The increasing demand for non-invasive methods has led in the last decade to close collaborations between computer scientists, mathematicians, conservators and wood scientists, resulting in improved imaging techniques such as X-ray computed tomography (CT) to allow non-invasive dendrochronological studies [12]. The first attempts to carry out dendrochronology on CT images date back to the 1980s [13, 14]. Although promising, those studies, as well as subsequent ones, failed to achieve an image quality good enough to enable measuring narrow tree rings (e.g. narrower than 0.8 mm) [15, 16]. They also illustrated how the variety in the anatomy of wood species and object shapes poses a challenge to the systematic application of CT for dendrochronological purposes. Wooden cultural heritage objects are often too large to perform regular CT imaging [17, 18], and high resolution is necessary to image the narrowest rings accurately [19]. Consequently, the object shape and dimensions coupled with the wood species may result in high requirements on the scanning system, as well as in long scanning times and large datasets [17, 20, 21]. [19] managed to obtain CT images from oak (*Quercus* sp.) and beech (*Fagus* sp.) test strips that reached a high resolution (24 μm) and allowed the observation of very narrow rings. This study was followed by a real breakthrough when [20] succeeded to date a collection of archaeological objects from the Viking era using dendrochronology on X-ray CT images. Since then, a few studies have demonstrated the successful implementation of dendrochronology based on CT imaging to date cultural heritage objects such as archaeological artefacts [22], Japanese Shinto sculptures [23], Norwegian late medieval sculptures and shrine-door panels[24], and a Netherlandish sculpture [25]. The results of those studies demonstrate the suitability of the method to visualize tree-ring patterns in archaeological wood with different degrees of preservation, and in historical polychromed wood from both broadleaf (*Quercus robur/petraea*) and conifer (*Chamaecyparis obtusa*) species.

Still, the scarcity of such studies contrasts with the growing demand for non-invasive dendrochronology. Achieving the required image resolution and quality for tree-ring research is still a challenge for most scanning facilities, in particular medical ones [26, 27]. Furthermore, the size of the object poses additional limitations that currently hinder the systematic application of this technique in the study of cultural heritage [12]. Therefore, our investigation of a painting on panel attributed to Rubens’ studio aimed at implementing X-ray CT scanning and achieving high-quality CT images to retrieve the tree-ring sequence in the wood and establish its date and provenance by dendrochronology. Our research delivered surprising results that should promote the improvement and systematic use of X-ray CT scanning to study historical art objects.

### The painting

**Fig 1:**
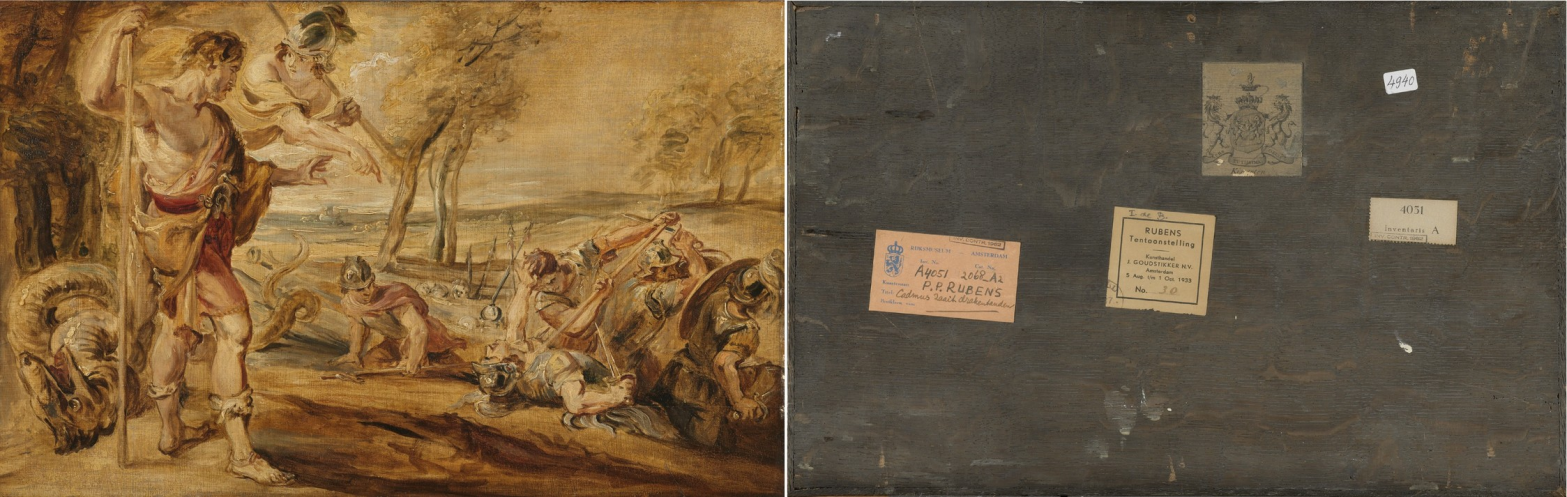
Front and back of the Rijksmuseum’s panel painting *Cadmus Sowing Dragon’s Teeth* (http://hdl.handle.net/10934/RM0001.COLLECT.5320). The strips of oak that surround the panel can be observed on the photo of the reverse.

The panel painting entitled *Cadmus Sowing Dragon’s Teeth* (hereafter *Cadmus*; Fig 1) is part of the collection of the Rijksmuseum in Amsterdam, The Netherlands (http://hdl.handle.net/10934/RM0001.COLLECT.5320). This small oil sketch on panel (27.7 cm height x 43.3cm width) is attributed to the studio of Peter Paul Rubens (1577–1640) and can be connected to another sketch of the same scene presently at a private collection, which is considered to be by Rubens himself, as well as to a large-size canvas painting of *Cadmus and Minerva*, now in the Prado museum in Madrid, Spain (181 cm x 300 cm, 1636–39, Prado, Madrid, P001713). Both oil sketches relate to a commission in 1636 to Rubens by Philip IV of Spain, to decorate the King’s hunting lodge Torre de la Parada near Madrid [28]. The full commission comprised 63 paintings depicting mythological themes. Although Rubens made the oil sketches for all mythological scenes, he appointed several other Flemish painters to execute many of the full-scale paintings. The canvas painting of *Cadmus and Minerva* from Torre de la Parada, and now at the Prado Museum, has been tentatively attributed to the Flemish painter Jacob Jordaens (1593–1678) [29]. The original oil sketch by Rubens is currently in a private collection in England. Technical art historical research on the *Cadmus* sketch at the Rijksmuseum was initiated to shed light on the attribution and purpose of this painting.

To determine the date and provenance of the wood used as support for the *Cadmus* sketch, dendrochronological research was first performed in 2007 by a dendrochronologist from the University of Hamburg. The panel is made of one single board of deciduous oak, 27.7 cm high by 43.3 cm wide. All four edges are covered with oak strips (Fig 1). This frame hampers the visualization of the tree rings on the transverse section, deeming the panel *a priori* unsuitable for dendrochronological research through traditional methods (i.e. measuring the tree rings on the transverse section at the edges of the boards). The dendrochronologist proceeded therefore with the research on the back of the panel, which represents the radial section of the wood. The last (most recent) ring in the panel was dated to 1637 and, accounting for missing sapwood rings and seasoning time of the wood, a plausible production date was proposed ‘from 1654 upwards’ [30]. That date placed the making of the painting after Rubens’ death in 1640, which would refute the connection of the sketch with the Torre de la Parada commission of 1636. Therefore, a dendrochronological re-examination of the panel was carried out by the same scientist in 2017. On this second occasion, the date of the outermost ring in the panel was established as 1591, estimating that ‘the creation is plausible from 1610 upwards’ [31]. Such disparity on the dates obtained in both occasions can be the result of faulty measurements due to the difficulty of measuring in the radial section of the panel. Although tree-ring research in the radial section of oak has been proven possible in exceptional cases [24, 32] it is a challenging endeavour in this case. The close proximity of the earlywood vessels of narrow rings hinders the discrimination of individual tree rings in some portions of the back of the panel, reducing the chances of obtaining a reliable tree-ring series. Therefore, although the date presented in 2017 was consistent with Rubens’ workshop activity, the disparity of the dates resulting from both dendrochronological analyses was still puzzling. In 2019, it was decided to re-examine the panel painting once again, this time using non-invasive X-ray CT imaging. This technology is available at the Centrum Wiskunde & Informatica (CWI) in Amsterdam (the Netherlands) and would allow the retrieval of a faultless tree-ring pattern from the transverse section of the panel.

## Materials and methods

### The FleX-ray Laboratory scanning facility

The FleX-ray Laboratory at the Centrum Wiskunde & Informatica (CWI) in Amsterdam was established in 2017 through the collaboration between CWI, TESCAN-XRE NV, Nikhef and Amsterdam Scientific Instruments (ASI) [33]. The laboratory offers use of the FleX-ray CT-scanner, as well as in-house reconstruction and image analysis techniques, managed and developed by the Computational Imaging group at CWI. The custom-built CT-scanner can accommodate challenging objects or scanning scenarios due to its full flexibility and highly adaptable control mechanism. The apparatus consists of a cone-beam microfocus X-ray point source and a flat panel detector of 1944-by-1536 pixels (14.5 cm wide by 11.5 cm high). Due to the space constraints of the cabin objects up to 50 cm^3^ can be imaged.

### Challenges of scanning a panel painting

A challenge specific to the scanning of a panel painting is due to the shape of the object. High power X-rays need to be employed to sufficiently penetrate substantial amounts of the material at certain angles (when the painting is perpendicular to the detector) but only a fraction of that material is in view at other angles (when the painting is parallel to the detector). This creates a sharp difference in the collected information with respect to the absorbed X-ray beam, leading to a dramatic contrast change in the reconstructed image. Furthermore, given that little is known about the possible damaging effects of radiation on oil paint, reducing the radiation dose and exposure time is a prerequisite when working with polychromed cultural heritage artifacts, leading to a trade-off between the image quality that can be obtained and the radiation dose [34]. Another factor that influences the absorption of the X-rays is the metal often present in the paint, which causes scattering of the X-ray photons, photon starvation, and beam hardening effects in the reconstructed images [35, 36]. Additionally, the size of the *Cadmus* painting (almost 44 cm in width) makes it necessary to perform multiple scans to image the entire object, and restricts the distance to the source and the detector, as the object needs to be rotated 360 degrees for each scan. All these factors impose limits on the image resolution that can be achieved.

**Fig 2:**
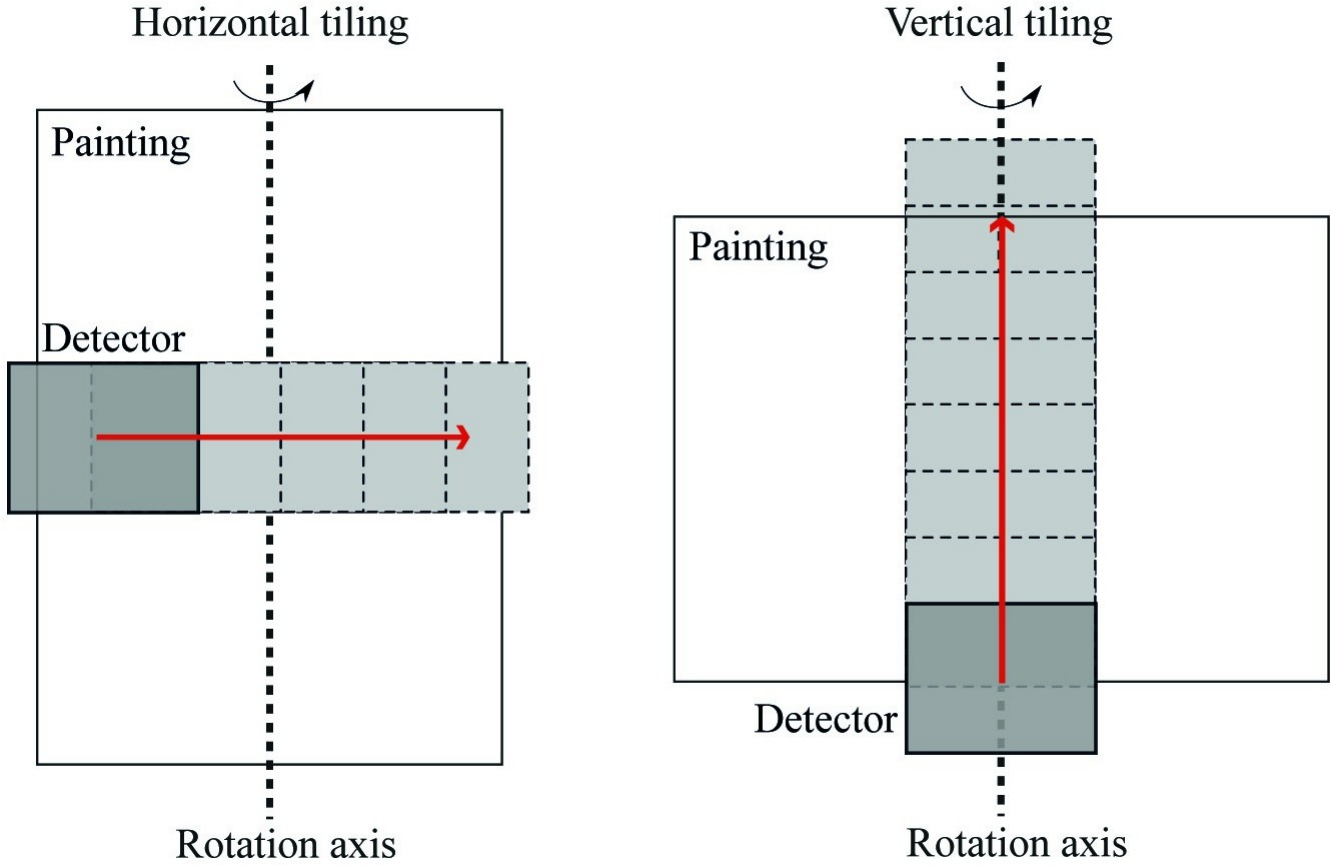
Possible orientations to scan the panel. Horizontal tiling (left): this setup requires fewer tiled scans, but the rotation axis lies outside the field of view of the detector for all tiles except the central one, resulting in parts of the painting to rotate in and out of the field-of-view. Vertical tiling (right): this setup requires more tiles, but the section of the painting we want to image stays within the detector field-of-view for all tiles during the rotation.

For dendrochronology, an image of the transverse section of the wood is needed, which means that a 2D slice of the 3D reconstruction is required. This is an advantage, as only a section of the object needs to be imaged. However, partial data in a CT scan presents challenges for the image reconstruction. Furthermore, there are two possible orientations to scan the panel (Fig 2): horizontal, with the painting supported on one of its sides, scanning from left to right; and vertical, with the painting supported on its top or bottom, scanning from bottom to top. To image a full cross-section of the object, several scans (henceforth referred to as tiles) must be performed with overlapping portions, adjusting the position of the source and detector each time between scans. In horizontal tiling this means that the centre of rotation does not remain within the detector view, and thus parts of the object rotate in and out of the field-of-view. In vertical tiling however, the slice we are interested in stays within the field-of-view during a full rotation. Taking this into consideration, we carried out a test scan with a mock board to establish the best set up and scanner settings for the painting (S1 Text).

### Scanning the painting and reconstructing the images

**Fig 3:**
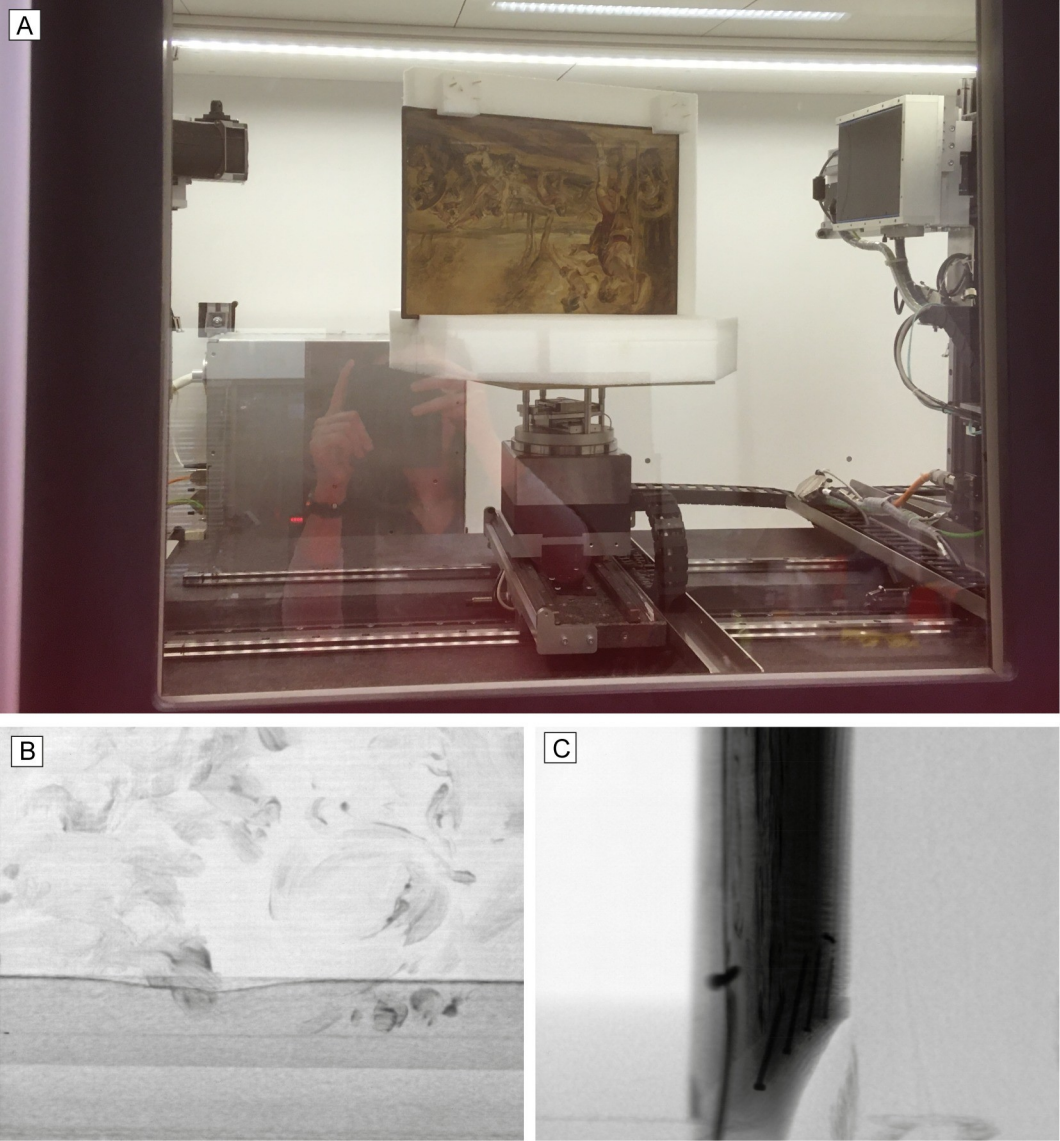
Scanning the *Cadmus* painting at the FleX-ray Lab. (A) Panel on the Ethafoam® mount placed on the rotation stage at approximately 90 degrees rotation. The vertical tiled scans are achieved by moving the source (on the left) and detector (on the right) up or down by the same distance, so that they stay directly opposite to each other; (B) X-ray image of Tile 2 at 0 degrees. The foam can be seen at the bottom, whereas the slightly darker area above represents the oak strip; (C) X-ray image of Tile 2 at 90 degrees. The X-rays must travel through approximately 44 times more wood in this direction. The nails that fix the oak strip to the panel can be seen.

The scan of the *Cadmus* panel was carried out in January 2020 in vertical-tiling orientation (Fig 3A) and following the workflow proposed by [37]. As only a small part stays in the view vertically, it is important to choose a region that contains as little metal as possible, i.e. no nails, because having them inside or in the surrounding of the field-of-view would cause inaccuracies within the reconstructed image. However, nails are not always visible on the outside of the frame. Therefore, to determine the best section to capture, an inspection of the X-ray images must be carried out prior to the scans. The panel was shifted slightly to the left of the mount to reduce the number of X-ray images affected by nails coming into the field-of-view.

Fifteen vertically tiled scans were performed to ensure enough overlap between sharply imaged sections. This was necessary to achieve a magnification of 2.0 on the X-ray images. Each tile consisted of 1600 X-ray images. The ratio of the longest to the shortest path that the X-rays travelled (i.e. width:depth) through the material was high, corresponding to about 44 times more material in one direction (Fig 3B and 3C). Consequently, the tube voltage and power were 70 kV and 70 mA for all tiles and a focal spot size of 22μm. A copper filter of 0.1cm thickness was used to reduce beam hardening. For the image reconstructions in this paper the ASTRA Toolbox [38] and FleXbox software [39] were used. The FDK reconstruction algorithm [40] was used after applying center of rotation correction. The reconstructed images were then put through a simple, 4-step post-processing routine using the open-source imaging software Fiji/ImageJ [41] (see S2 Text for further details).

### Dendrochronological research on the CT images

Tree rings were measured on each tile separately using CooRecorder & CDendro package v. 9.0.1 [42]. The number of overlapping rings between consecutive images was registered, so that the tree-ring series of each tile could be matched visually with its neighbours and merged into an average curve afterwards. The tree-ring series obtained was compared with the network of historical reference chronologies of oak available for areas in central, eastern and northern Europe that supplied oak for art objects in western Europe [4, 6, 10], which represent Eastern France (Tegel, unpublished), Germany (Tegel, unpublished; [43, 44]), Western Sweden [10, 45] and the south eastern Baltic including Poland [4, 46, 47]. Crossdating was carried out with PAST4 v. 4.3.1025 [48] and followed standard dendrochronological procedures for European oak [49, 50] (see S3 Text for details).

## Results

### A slice through the painting: The CT reconstructed images

**Fig 4:**
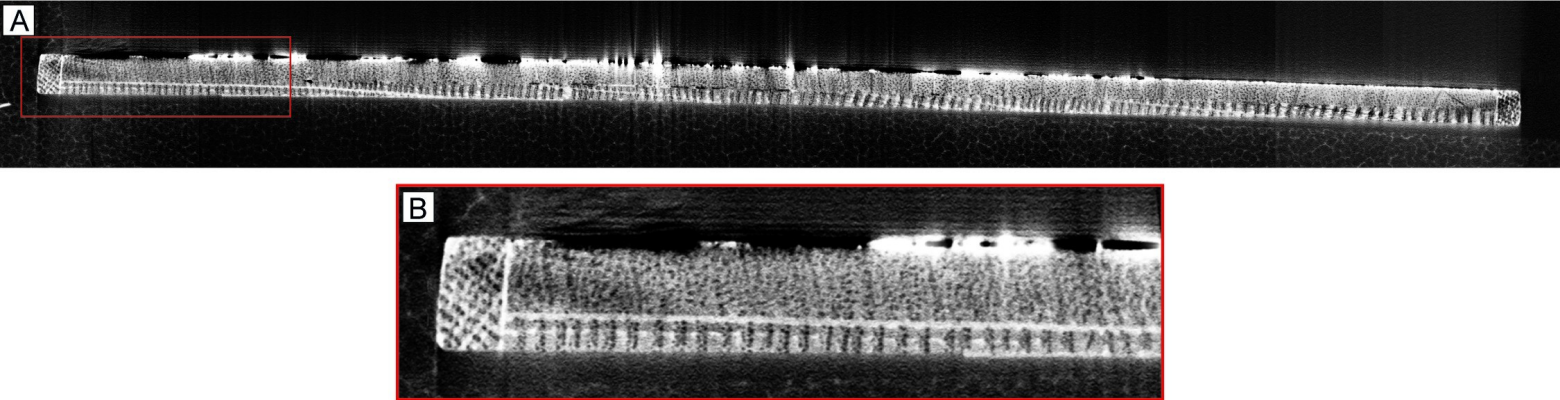
Reconstructed CT images. (A) Reconstructed CT image of the full cross-section of the panel, providing an overall impression of the configuration of the panel and the transverse section of the oak back board, which has been processed radially from the parental tree; (B) reconstructed CT image of the Tile 2. Tile 2 corresponds to the left edge of the panel in (A), and the reconstructed image (with 37.3 micron resolution) shows: the oak strip on the left; the paint layer on the upper part, with white areas caused by highly absorbing metal-containing pigments in the paint; the original wooden painting support, which appears to be of a diffuse-porous species; and a second (oak) board glued on the back of the original board. The oak board was initially the target of this investigation as it was assumed to be the original support. Tree rings in the oak board are evident in this image (growth direction towards the left in both images).

Fifteen CT scans (tiles) were necessary to cover the entire height of the painting with optimal overlap between them. The X-ray CT data allowed the reconstruction of a slice of the painting with a resolution of 37.3 micron (Fig 4A). The paint layer contains metal-based pigments such as lead white, which produced shiny (white) spots in the reconstructed image, but did not preclude the visualization of the wood. When examining the reconstructed image of a tile from one edge of the painting, the narrow oak strip becomes evident (Fig 4B). Moreover, the image shows that the panel is composed of two thin boards glued together. The oak board, visible at the back, which was the target of this dendrochronological study, is 2.5 mm thick, whereas the board used as the original painting support, is 4.5 mm thick and is made of some type of diffuse-porous wood species.

### Identification of the diffuse-porous species

The realisation that the original painting support is a diffuse-porous species motivated the identification of the wood, as this may provide a clue about the place where the painting was made. Identification of wood species is traditionally done by observation of key (diagnostic) anatomical features in thin sections obtained from the transverse, radial, and tangential sections of the wood. Some species show distinct anatomical features in the transverse (cross-) section that make them distinguishable by the naked eye. Such is the case, for example, for deciduous oaks, which show large earlywood vessels placed in a ring-porous disposition and large multiseriate rays [51]. Diffuse-porous species are more difficult to discriminate by observing the transverse section, and visual access to the tangential and radial sections is usually also needed. In this case however, the resolution of the CT images corresponding to those sections was not enough to allow the identification of the wood from the front board.

**Fig 5:**
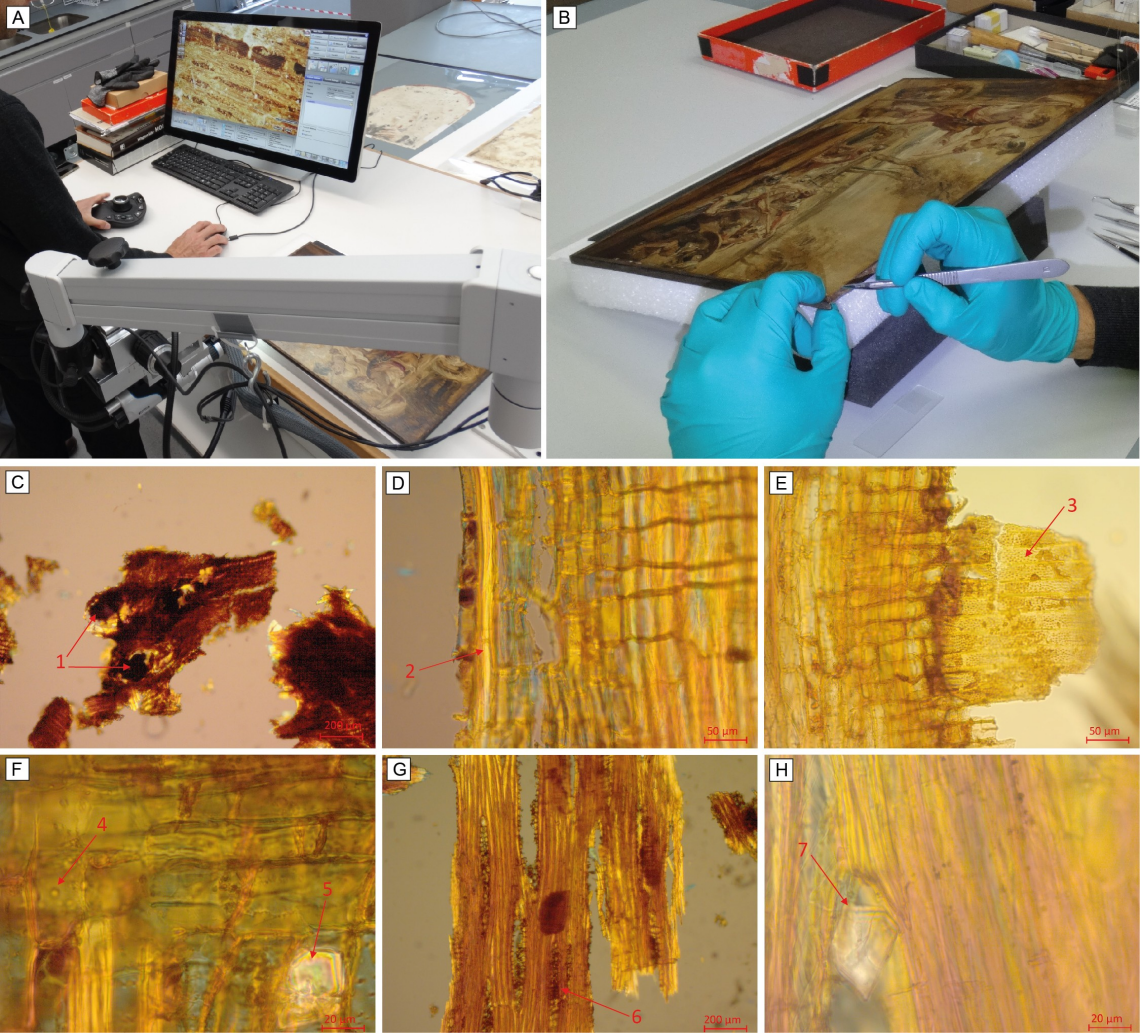
Species identification of the front (original) support. (A) direct observation of wood anatomical features with the Hirox RH-2000 digital microscope; (B) removal of a micro sample of wood from the original support in the top-right corner for wood identification. Key anatomical features characteristic for *Swietenia* sp. [52–54]: (C) Transverse section, 50x: large vessels 100–270 μm, diffuse-porous, mostly solitary, occasionally in pairs; gums in vessels (1); parenchyma apotracheal and paratracheal; terminal parenchyma are not visible due to small sample size. (D) Radial section, 200x: septate fibres (2) present; gums in parenchyma. (E) Radial section, 200x: Simple perforation plates; numerous vessel ray pits (3) alternate, simple, very small 1.9 to 3.5μm. (F) Radial section, 500x: cross-field pits small and bordered (4), prismatic crystals in marginal (procumbent) ray cells (5). (G) Tangential section, 200x: Rays 1–4 cells wide (6), 9–20 cells high, slightly heterogeneous, slightly storied. (F) Tangential section, 500x: prismatic crystals in marginal (procumbent) ray cells (7).

Upon closer examination of the oak strips surrounding the panel, we observed a small crack at about 12 cm from one of the corners. The fragment was held in place by just one nail (with no glue on that part of the strip), so we proceeded to its careful removal in order to have direct access to a portion of the original panel for the identification of the wood species. Direct observation with a 3D digital microscope (Hirox RH-2000 Digital Microscope) was carried out on the exposed section of the wood panel (Fig 5A) but was insufficient to reach a conclusive identification. Therefore, we decided to take micro samples for examination under a transmitted-light microscope. A scalpel was used to collect micro thin sections from the exposed upper right corner of the board, without touching the paint layer (Fig 5B). They were examined under a Zeiss Axiolab A1 microscope equipped with a ZEISS Axiocam 105 camera. Image processing was conducted using Zen2 Imaging Software. Diagnostic anatomical features were checked against several anatomical keys for identification of wood species [52–55]. This time, we could observe key anatomical features such as gum deposits in heartwood vessels and prismatic crystals located in marginal (procumbent) ray cells, which not only discarded some European diffuse-porous species historically used in painting workshops (e.g. poplar (*Populus* sp.), walnut (*Juglans regia*), beech (*Fagus sylvatica*)), but that also pointed towards some tropical species from the *Meliaceae* family. Comparing more detailed key anatomical features observed in our micro-sample with photos of those characteristics openly available in InsideWood [55] and DeltaIntkey we narrowed down the identification to *Swietenia* sp. (including *Swietenia macrophylla* an *Swietenia mahagoni*) and *Cedrela* sp. (including *Cedrela odorata*). *Swietenia* is a genus distributed in seasonally dry, lowland neotropics from Mexico to Brazil and Bolivia [56, 57] and is described as diffuse-porous species [55]. *Cedrela* is distributed in tropical to sub-tropical areas from southern Mexico to northern Argentina, and has a very diverse porosity (ranging from ring-porous to diffuse porous) depending on the geographical area [58]. Consequently, vessel distribution cannot be taken as a diagnostic characteristic for this species. A key characteristic that discriminates both species is the intervessel pit diameter, which is much smaller in *Swietenia* than in *Cedrela*. While the small size of our sample did not allow the identification of this feature, were able to observe rather wide (up to four cells) multiseriate rays, and could also identify and measure small vessel ray pit diameters ranging from 1.9 to 3.5 μm, pointing both characteristics towards *Swietenia*, rather than *Cedrela* (Fig 5C–5H). Therefore, the original panel is most likely made of *Swietenia* sp.

### Dendrochronological dating and wood provenance

**Fig 6:**
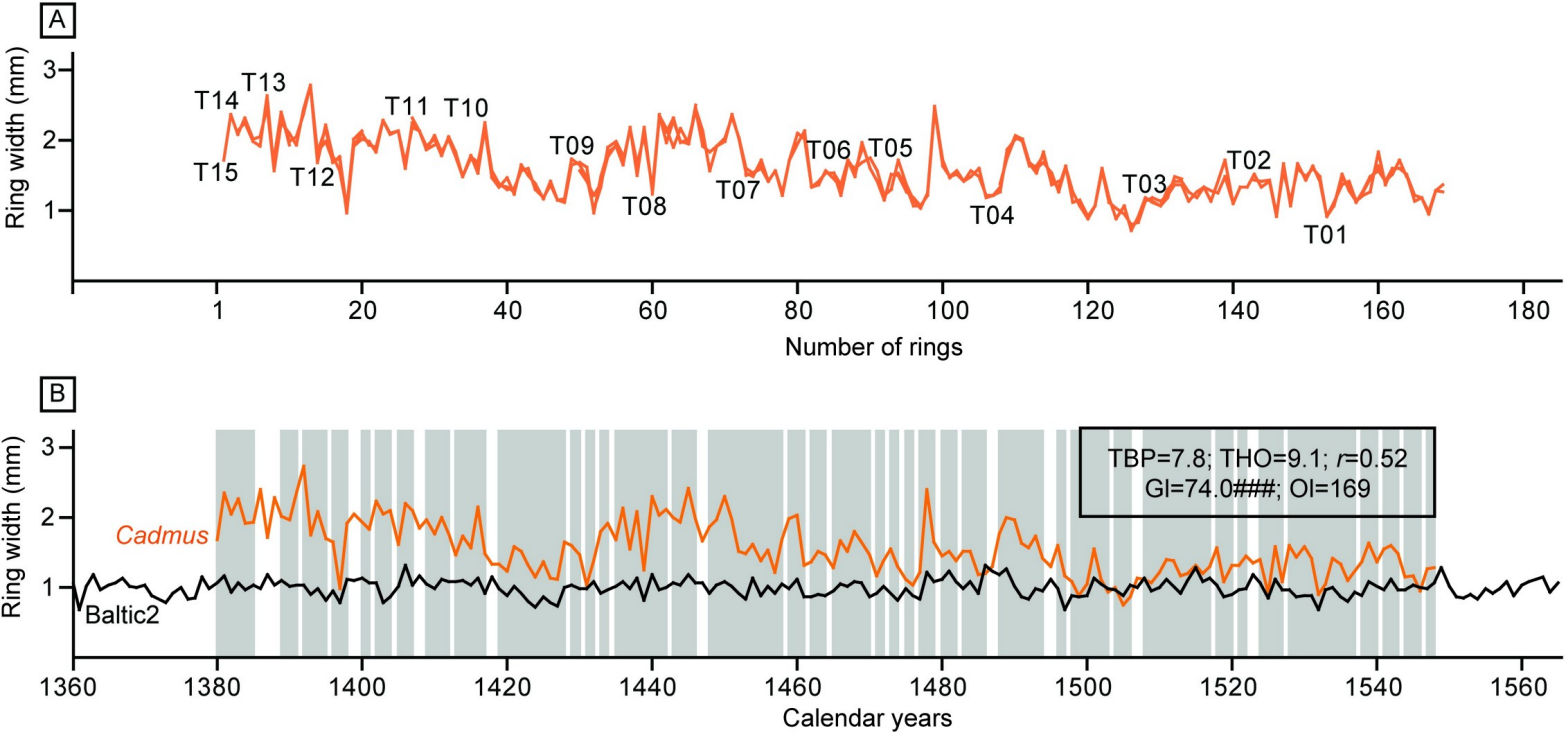
Results of the dendrochronological research. (A) Cross-match between the tree-ring series obtained from each individual tile. (B) Absolute dating of the mean curve obtained from averaging the measurements of the individual tiles (*Cadmus*) crossdated with the Baltic2 chronology. TBP, Student’s *t*-value as implemented by [60] for tree-ring studies; THO, Student’s *t*-value as implemented by [43]; *r*, correlation coefficient; Gl, percentage parallel variation between the overlapping portion of the compared tree-ring series [61], accompanied by its signification level; Ol, overlap (see S3 Text for details about the statistical tests used).

Tree rings of the oak board were clearly visible in the CT images and a 169-year-long series was assembled by merging the measurements of the individual CT images (Fig 6A; see Table A and B in S1 Table). Crossdating with reference chronologies resulted in the dating of the outermost ring present in the panel to the year 1548 C.E. The best match was obtained with the Baltic2 chronology assembled by [4] (Fig 6B). This chronology represents the south-eastern Baltic, therefore the wood from the back panel originates from that area, possibly Poland. The lack of sapwood in this oak board hampers estimating the felling date of the tree, hence only a *terminus post quem* date (i.e. date after which the tree was cut) can be provided. Given the provenance of the wood in the south-eastern Baltic, and the available sapwood estimates for Poland [46, 59], it is possible to estimate within a 90% confidence interval that the tree was cut after 1557 C.E.

Tree rings on the original tropical board are unfortunately not distinct enough in the CT image to obtain a tree-ring series. Furthermore, existing chronologies of *Swietenia* species in South America barely reach the late 19th century [62]. Therefore, dendrochronology could have not served to date this panel even when a tree-ring series could have been retrieved from the CT images.

## Discussion

### Non-invasive research of panel paintings through X-ray CT images

State-of-the-art X-ray CT scanning has seldom been used on panel paintings for dendrochronological research. Two studies report on the potential of this technology to assess the manufacturing technique and preservation state of panel paintings [26, 63], whereas only [24] presents a successful dendrochronological study of two panel-doors from a shrine in Norway. In our study, the X-ray CT image was key to discover the two different boards and to allow the correct dating of the oak one. Our dendrochronological results refute the previous dating reports, demonstrating that retrieving tree-ring series from the radial to radial/tangential section in the back of oak panels, although a non-invasive procedure in itself, can potentially lead to measuring errors [24].

This research shows that laboratory X-ray CT scanners can reach the resolution needed to perform dendrochronological research on panel paintings. However, the image quality may be affected by “grain noise” the smaller the wood features are [19]. Although this should not be a problem for visualizing tree-rings of oak species (the large size of earlywood pores disposed along the ring boundary facilitate the identification of individual tree rings), it may pose a challenge to identify ring boundaries in diffuse-porous species such as poplar, walnut, or as in this study, tropical species. In this type of species, pores and vessels are usually distributed across the ring width, and the small parenchyma cells making up the ring boundaries might not be rendered sharply even at high resolutions (e.g. 25μm). This is the reason why the tree rings of the tropical board are undistinguishable in the CT image.

We have also investigated the challenges presented by the shape of the panel for CT scanning. For this particular panel we found that acquiring the X-ray images in a “vertically tiled” mode on the landscape orientation provided excellent results, but it required several tiles, making it a time-consuming procedure. A larger detector would reduce the number of tiles needed. Given the available state-of-the-art detectors, the compromise however would be lower resolution. The optimal scanning routine may be different for other panels, as the paint material, the length of the panel, the size of the detector, the focal spot size of the tube and the space constraints of the X-ray facility are factors that influence the range of motion and reconstructed image quality. Further, monochromatic X-ray beams or polychromatic X-ray beams with higher photon count (higher tube voltage and power settings) such as those available at synchrotrons could result in less noisy X-ray images with reduced imaging artefacts [64, 65].

### Double panelling in painting supports

Painting supports consisting of a double panel can derive from conservation treatments such as a partial transfer. In this 19th century conservation procedure the panel was thinned, but not totally removed (full transfer), and subsequently glued to another wooden board. Different recommendations were issued on the subject. According to [66], 19th century restorers recommended “gluing the original panel […] to a very old oak board” [67] and that “the original panel should not be thicker than 3–6 mm” [68]. Weakened original wooden supports, damaged for example by wood worms or cracking, were sometimes thinned and glued onto another board of solid wood, plywood, or from the 1920s, onto Masonite boards [66].

There could also be more economic, profitable reasons for double panels. Up to the late 19th century, some double-sided panel paintings were separated by sawing the boards longitudinally with veneer frame saws or by splitting, so that both paintings could be exhibited or sold separately [66]. The remaining supports would be so thin that they would be highly reactive to changes in temperature and relative humidity, requiring some type of stabilization procedure such as cradling, or indeed attachment onto another wooden support [66]. Although *Swietenia* is a rather stable type of wood [58], it is possible that the oak panel was added during a conservation treatment. The thickness of the original *Swietenia* support in the *Cadmus* sketch is just 4.5 mm, which suggests that thinning of the panel could have happened prior to attaching it to an oak board. The dendrochronological date of the auxiliary oak support of the *Cadmus* sketch (1548), pre-dates by far the commissioning date of 1636. Since it is not possible to estimate how much wood and tree rings were removed during the preparation of the oak board, we cannot know whether the felling of the tree occurred before or after 1636. It is possible that the oak board was already applied in the 17th century, after the sketch was finished, using a contemporary oak board trimmed to size. However, it could also have been done at any time between then and the late 19th—early 20th century, before it entered the Rijksmuseum collection.

Another possibility is that the tropical panel was covered with the oak board and oak strips along the edges for deceptive rather than conservation purposes, to give the impression that the sketch was painted on oak, which was ubiquitous in Netherlandish painting practice at the time [9]. On the X-ray images we could observe that the oak strips are all attached with what seem to be 19th-century wire drawn nails like the one retrieved after removing the loose strip fragment. However, the strips could have also been added for the purpose to help framing the panel, so that the frame would not cover up the picture. Ongoing analytical and technical art historical research shall provide further clues to confirm or discard these hypotheses.

### Use of tropical species as support for panel paintings

The tropical wood of the original support attests the global character of 17th century trade, and demonstrates the use (or reuse) of exotic species in Flemish studios. Species such as *Swietenia mahagoni* and *Cedrela odorata* originating from (sub)tropical America arrived in Europe already in the 16th century through Seville (Spain) [69, 70]. Since the late Middle Ages, Seville had become the major trade-hub of southwestern Europe, and Dutch, Flemish and German-Hanseatic merchants traded all kind of products back and forth, making for instance Swedish oak, Baltic wainscots and Scandinavian pine available in the Iberian city [10, 69]. It is therefore not surprising that in such a context of global trade these tropical woods made it back to the Low Countries aboard Dutch and Flemish vessels, either as timber or as processed boards, or even as boxes transporting other commodities such as sugar [71]. [9] report six paintings by Rembrandt made on *Swietenia mahagoni* between 1634 and 1654, which provide evidence of the availability of this species in the Low Countries at the time of the commission for the *Torre de la Parada*. Consequently, it is plausible that the *Cadmus* painting was made in Antwerp in the 1630s. *Swietenia* sp. has also been found as support for a painting by Dutch artist Aelbert Cuyp (1620–1691) [72]. In the Dendro4Art database of the Netherlands Institute for Art History, which contains almost 6,000 entries of dendrochronological inspection and research of panel paintings (including wood identifications), *Swietenia* is reported for only seven additional paintings, three by the Dutch artists Nicolaas Verkolje (1673–1746), Hendrik Meijer (1744–1793) and Johannes Warnadus Bilders (1811–1890), and four by 19th century artists, such as French painters Narcisse Virgile Diaz de la Peña (1807–1876) and Constant Troyon (1810–1865), and Belgian painter Alfred Stevens (1823–1906) [73]. The overall scarcity of examples, in particular on 17th century works, points towards a reuse of this timber, and hampers making further inferences about its specific selection to be used as support for paintings, or about the place where the *Cadmus* sketch was made.

### Implications of double panelling for dendrochronological studies

CT images may reveal unexpected results. The discovery that the *Cadmus* painting was originally executed on a panel of tropical wood, which was glued onto an oak board at some point in time, has opened new art historical enquiries that will be pursued in further studies. Furthermore, it raises the question of how many other double panels may have gone unnoticed. From a dendrochronological perspective, research on panel paintings glued onto a new oak support may lead to a very different date if the double panelling is not identified. The back panel may pre- or post-date the date of the artist’s death, thereby triggering questions about authenticity, possible re-use of materials and so on. X-ray CT imaging is therefore a powerful tool to assist in dendrochronological research of wooden objects.

### Towards the systematic application of CT in material heritage studies

This research demonstrates the valuable contribution of CT scanning to the study of panel paintings. Unlike medical CT scanners, laboratory ones like the one used in this study, have the capacity to produce the high-resolution images needed to measure tree rings accurately, providing the ultimate non-invasive method to retrieve the tree-ring patterns from wooden artefacts. Furthermore, this technology allows the selection of the most optimal slice(s) for the research. In the case of panel paintings, the slices where the surface paint contains less metals are the most desirable ones, as those will provide the highest quality of the reconstructed image. In polychrome sculptures for example, several slices can be selected at different heights, where protuberances in the design may provide more tree rings towards the center and the outside of the tree, allowing to retrieve the longest possible tree ring series from the object [25].

Future investigations of panel paintings with CT should also consider other scanning geometries and energy settings that might be more suited for objects with elongated shapes. Increasing energy may serve to obtain better images from elongated objects, but the short- and long-term effect of X-rays on pigments and preparatory layers has yet to be studied. Carrying out further testing will allow the development of protocols to improve image quality and image processing time while accounting for the shape and materials.

## Supporting information

S1 Text (PDF)

S2 Text (PDF)

S3 Text (PDF)

S1 Table (PDF)
