# Supplementary material for: X-ray computed tomography for non-invasive dendrochronology reveals a concealed double panelling on a painting from Rubens’ studio
Source: PLoS One. 2021 Aug 27;16(8):e0255792. doi: 10.1371/journal.pone.0255792 (PMC8396786; doi:10.1371/journal.pone.0255792)
Supplement: S1 Text — (PDF) [file pone.0255792.s001.pdf]

S1 Text. Testing with a mock plank

Since the shape of panels in general poses a challenge for CT reconstructions due to the length-to-width ratio, we carried out test-scans with an oak mock plank provided by furniture conservators of the Rijksmuseum. The plank had the same dimensions as the *Cadmus* painting, and was also covered on one side by a preparatory layer.

To reduce any inaccuracies due to a tilt of the plank, it had to remain in upright position at all times. To this end, we designed and built a mount made of Ethafoam®, which only lightly absorbs the X-rays and does not influence the reconstruction results. The plank was placed into a slit at the base of the mount and was secured to the upright position with small pieces of foam (**Fig. S1A**). For the real panel these pieces would only touch the frame.

When the object is perpendicular to the detector, one side of the panel is closer to the source and is magnified greatly. The consequence is that only a small region in the middle of the reconstructed image is sharp and therefore a large overlap in the tiles is necessary to capture the entire object in focus.

Tests with varying numbers of tiles (adjusting the position of the source and detector to a different vertical position each time between scans) and image resolution were carried out (**Fig. S1B**). The end grain (transverse surface) of this mock plank was also cleaned with Stanley® knives and photographed using a compact camera in macro mode (**Fig. S1C**). The tree ring-widths were measured in the digital photos with the CooRecorder & CDendro package v. 9.0.1 (Cybis Elektronik & Data AB) in order to have a reference measurement from the plank to allow cross-validation of the tree rings measured on the CT image.

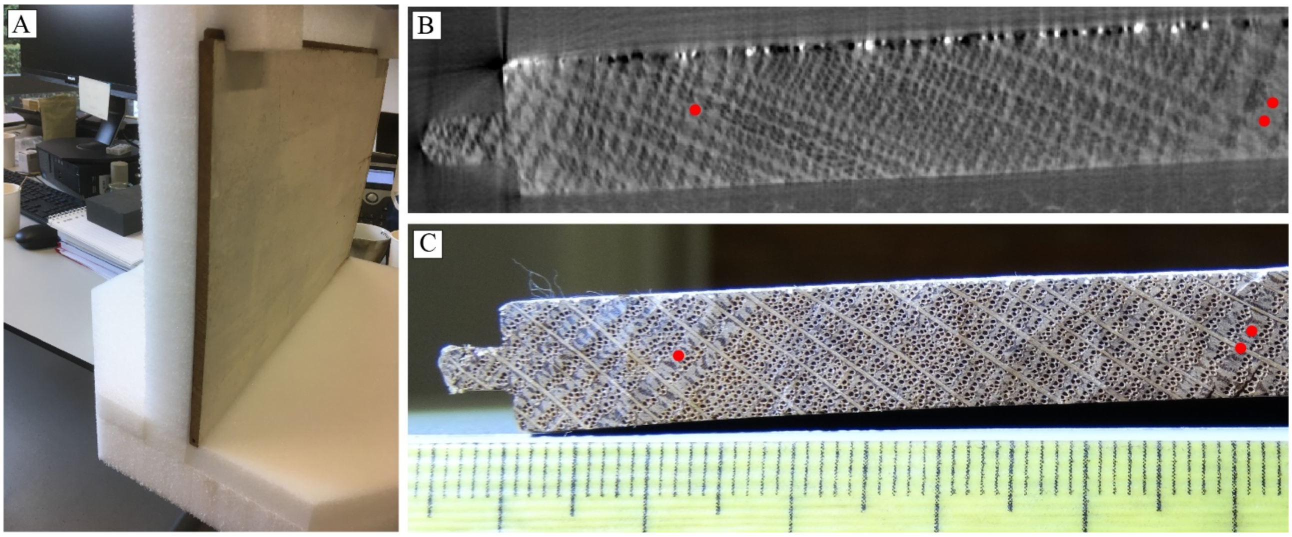

S1 Fig. Mounting of the mock plank for vertical tiling at the FleX-ray Laboratory. (A) The test plank on the Ethafoam® mount. (B) Reconstructed image of one tile from the upper part in the centre of the plank. (C) transverse surface of the corresponding upper portion of the section reconstructed in (B), where it has been cleaned with Stanley® knives to allow visualization of the tree-rings. The dots in (B) and (C) indicate corresponding tree rings.
