# Supplementary material for: X-ray computed tomography for non-invasive dendrochronology reveals a concealed double panelling on a painting from Rubens’ studio
Source: PLoS One. 2021 Aug 27;16(8):e0255792. doi: 10.1371/journal.pone.0255792 (PMC8396786; doi:10.1371/journal.pone.0255792)
Supplement: S2 Text — (PDF) [file pone.0255792.s002.pdf]

S2 Text. Reconstruction and post-processing of the images

Images were reconstructed tile by tile using the ASTRA Toolbox (1) and the FleXbox software (2). The FDK reconstruction algorithm (3) was used after applying center of rotation correction. The reconstructed images were then put through a simple, 4-step post-processing routine using an open-source imaging software Fiji/ImageJ (4). The following steps were performed on the individual tiles, in the given order: i) the object was enclosed in a rectangle, where the contrast was enhanced best fit for this section (accessed by Process > Enhance Contrast); ii) the saturated pixel parameter was set to 1% with the histogram equalized; iii) the resulting image was further cleared up with de-speckle which removes random noise (Process > Despeckle); and then iv) sharpened and smoothed using the inbuilt functions (Process > Sharpen and Process > Smooth). The final image was saved as a TIF file. To obtain an image of the full cross section, the images of the separate tiles were then merged using the FleXbox software.

References

1. van Aarle W, Palenstijn WJ, Cant J, Janssens E, Bleichrodt F, Dabravolski A, et al. Fast and flexible X-ray tomography using the ASTRA toolbox. Opt Express. 2016;

2. Kostenko A, Palenstijn WJ, Coban SB, Hendriksen AA, van Liere R, Batenburg KJ. Prototyping X-ray tomographic reconstruction pipelines with FleXbox. SoftwareX. 2020;11:100364.

3. Feldkamp LA, Davis LC, Kress JW. Practical cone-beam algorithm. J Opt Soc Am A. 1984;1(6):612–9.

4. Rueden CT, Schindelin J, Hiner MC, DeZonia BE, Walter AE, Arena ET, et al. ImageJ2: ImageJ for the next generation of scientific image data. BMC Bioinformatics. 2017;18(1):529.
