# Supplementary material for: X-ray computed tomography for non-invasive dendrochronology reveals a concealed double panelling on a painting from Rubens’ studio
Source: PLoS One. 2021 Aug 27;16(8):e0255792. doi: 10.1371/journal.pone.0255792 (PMC8396786; doi:10.1371/journal.pone.0255792)
Supplement: S3 Text — (PDF) [file pone.0255792.s003.pdf]

S3 Text. Dendrochronological analysis

Tree rings were measured as coordinates on each individual tile and subsequently converted to Heidelberg format with the software package CooRecorder & CDendro v. 9.0.1 April 19 2017 (Larsson, Cybis Elektronik & Data AB). The number of overlapping rings between the tiles were written down. The 15 measurements in Heidelberg format were uploaded into PAST4 v.4.3.1025 and crossdated visually. Subsequently, they were averaged into the tree-ring series *Cadmus* containing 169 rings. *Cadmus* was compared with reference chronologies of oak from central, northern and eastern Europe using the PAST4 commands Assistants>Multisync>Find best matches>Show the best 1 position, without applying thresholds and setting the TTest Algorithm as Exact. The output was exported into Excel and order from higher to lower values by best TBP. The following statistical tests are calculated by PAST4 v. 4.3.1025 and were considered to identify the date:

- Student's *t* test applied after transforming the data according to (1) (TBP): in this transformation a five-year moving average is run for both sample and reference prior to calculating the correlation coefficient. The standardized (dimensionless indexed series) are then used to calculate the correlation coefficient and subsequently the *t*.

- Student's *t* test applied after transforming the data according to (2) (THO): this transformation applies a logarithmic detrending to the series and reference, and the resulting values are used for subsequent calculation of correlation coefficient and *t* value.

TBP and THO values above 6 for overlaps over 100 rings are typically indicative of a correct match.

- Gleichlaufigkeit (GL) as described by (3). This non-parametric test calculates, for the overlapping period between two tree-ring series, the percentage of years in which the ring-widths increase (+1) and decrease (-1) in synchrony, considering also the years with no variation (0).

- Statistical significance of the GL (SL) considers the overlapping portion and is expressed as # ($p<0.05$), ## ($p<0.01$), and ### ($p<0.001$).

GL values higher than 65%, for an overlap of 100 rings are highly significant, but should always be used in combination with high *t* values to identify the match.

For specific formulas refer to the PAST4 user´s manual (© 1995-2011 by SCIEM / DI Bernhard Knibbe).

References

1. Baillie MGL, Pilcher JR. A simple crossdating program for tree-ring research. Tree-Ring Bull. 1973;33:7–14.

2. Hollstein E. Mitteleuropäische Eichenchronologie. Mainz am Rhein: Verlag Phillipp von Zabern; 1980. 273 p.

3. Eckstein D, Bauch J. Beitrag zur Rationalisierung eines dendrochronologischen Verfahrens und zu Analyse seiner Aussagesicherheit. Forstwissenschaftliches Cent. 1969;88:230–250.
