# Supplementary material for: X-ray computed tomography for non-invasive dendrochronology reveals a concealed double panelling on a painting from Rubens’ studio
Source: PLoS One. 2021 Aug 27;16(8):e0255792. doi: 10.1371/journal.pone.0255792 (PMC8396786; doi:10.1371/journal.pone.0255792)
Supplement: S1 Table — (PDF) [file pone.0255792.s004.pdf]

Table A. Crossdating overlaps between the tree-ring series obtained from the individual tiles and resulting statistical values. Ol: overlap.

**Sample**	**Reference**	**Ol**	**Gl**	**SL**	**TBP**	**THO**
T01	T02	16	78.1	#	5.18	4.63
T02	T03	22	90.9	###	9.91	9.49
T03	T04	12	75.0	#	3.66	3.90
T04	T05	26	76.9	##	8.68	7.07
T05	T06	23	82.6	###	12.90	9.86
T06	T07	20	90.0	###	7.34	7.02
T07	T08	17	82.4	##	8.06	6.79
T08	T09	17	97.1	###	8.61	4.96
T09	T10	17	91.2	###	8.04	8.96
T10	T11	22	75.0	##	11.00	8.50
T11	T12	16	90.6	###	12.60	8.26
T12	T13	17	97.1	###	12.50	8.40
T13	T14	13	80.8	#	8.67	5.82
T14	T15	8	81.3	#	3.99	2.76

Table B. Dendrochronological results of the individual tiles and the full cross-section (*Cadmus*). RW: ring width.

**Keycode**	**Portion scanned**	**Species**	**Length**	**StartYear**	**EndYear**	**Min. RW Value (mm)**	**Max. RW Value (mm)**	**Mean RW Value (mm)**	**Std.Dev. (mm)**
T01	Tile 1	QUSP	16	1533	1548	0.97	1.57	1.31	0.19
T02	Tile 2	QUSP	27	1522	1548	0.9	1.79	1.32	0.22
T03	Tile 3	QUSP	36	1508	1543	0.9	1.68	1.32	0.20
T04	Tile 4	QUSP	33	1487	1519	0.79	2.02	1.30	0.30
T05	Tile 5	QUSP	40	1473	1512	0.71	2.43	1.35	0.34
T06	Tile 6	QUSP	32	1464	1495	1.05	2.39	1.51	0.29
T07	Tile 7	QUSP	32	1452	1483	1.01	2.4	1.51	0.27
T08	Tile 8	QUSP	30	1439	1468	1.2	2.38	1.74	0.34
T09	Tile 9	QUSP	27	1429	1455	1.19	2.4	1.79	0.33
T10	Tile 10	QUSP	32	1414	1445	0.94	2.45	1.64	0.38
T11	Tile 11	QUSP	30	1406	1435	0.99	2.28	1.60	0.33
T12	Tile 12	QUSP	29	1393	1421	0.94	2.24	1.77	0.31
T13	Tile 13	QUSP	23	1387	1409	1.01	2.73	1.96	0.33
T14	Tile 14	QUSP	18	1382	1399	1.01	2.73	1.98	0.39
T15	Tile 15	QUSP	10	1380	1389	1.68	2.35	2.07	0.21
**Cadmus**	**Full cross-section**	**QUSP**	**169**	**1380**	**1548**	**0.75**	**2.73**	**1.56**	**0.39**
